# Cardiac amyloidosis presenting as refractory heart failure: Role of GLS in diagnosis and on follow up

**DOI:** 10.21542/gcsp.2026.7

**Published:** 2026-02-28

**Authors:** Chigullapalli Sridevi, Susheel Kumar Malani, Prakyath Shetty

**Affiliations:** Department Of Cardiology, Dr. DY Patil Medical College, Pune, Maharashtra, India

## Abstract

**Background:** Cardiac amyloidosis represents a challenging diagnosis in patients with multiple myeloma, often presenting as refractory heart failure with subtle early manifestations that can delay appropriate treatment.

**Case:** We report a 55-year-old male with a three-year history of multiple myeloma who presented with progressive dyspnea and bilateral lower limb edema. Despite being normotensive and non-diabetic, he exhibited clinical signs of volume overload with elevated jugular venous pressure and bilateral crepitations. Electrocardiography revealed left ventricular hypertrophy with strain pattern, left axis deviation, and left bundle branch block, raising suspicion for infiltrative cardiomyopathy. Transthoracic echocardiography demonstrated moderate concentric left ventricular hypertrophy with reduced ejection fraction (40%) and severe grade III diastolic dysfunction. Global longitudinal strain analysis revealed markedly reduced GLS at −9.7% with characteristic apical sparing pattern, creating the pathognomonic “cherry-on-top” appearance. Despite optimal heart failure management and continued multiple myeloma treatment, six-month follow-up demonstrated dramatic GLS deterioration to −1.9% , indicating progressive cardiac amyloidosis and poor prognosis.

**Conclusion:** Global longitudinal strain serves as both a sensitive diagnostic tool and powerful prognostic marker in cardiac amyloidosis secondary to multiple myeloma, supporting its integration into routine cardiac surveillance protocols for patients with plasma cell disorders to enable earlier diagnosis and better risk stratification.

## Introduction

Cardiac amyloidosis represents a progressive infiltrative cardiomyopathy characterized by the extracellular deposition of misfolded protein fibrils within the myocardium, leading to restrictive heart failure and significant morbidity and mortality^1^. Among the various types of systemic amyloidosis, light chain (AL) amyloidosis associated with plasma cell disorders, including multiple myeloma, accounts for approximately 70% of cardiac amyloidosis cases and carries the worst prognosis with median survival of 4–6 months when untreated^2,3^.

The clinical presentation of cardiac amyloidosis often mimics other forms of heart failure, making early diagnosis challenging yet crucial for optimal patient outcomes^4^. Traditional echocardiographic parameters frequently fail to detect early cardiac involvement, as wall thickness and ejection fraction may remain preserved until advanced stages of the disease^5^. Global longitudinal strain (GLS) has emerged as a sensitive and specific tool for early detection of cardiac amyloidosis, with characteristic apical sparing pattern demonstrating superior diagnostic accuracy compared to conventional echocardiographic measures^6,7^. This non-invasive biomarker not only aids in early diagnosis but also serves as a valuable tool for monitoring treatment response and disease progression.

Multiple myeloma, a malignant plasma cell disorder, is associated with AL amyloidosis in approximately 10–15% of cases, with cardiac involvement occurring in 50–70% of patients with AL amyloidosis^8^. The coexistence of these conditions presents unique diagnostic and therapeutic challenges, as symptoms of heart failure may be attributed to other causes related to the underlying hematological malignancy or its treatment^9^.

We present a case of cardiac amyloidosis manifesting as refractory heart failure in a patient with multiple myeloma, highlighting the diagnostic utility of GLS and its role in clinical management and follow-up monitoring.

## Case presentation

A 55-year-old male patient presented to the cardiology outpatient department with a one-month history of progressive dyspnea on exertion and bilateral lower limb swelling. The patient had a significant medical history of multiple myeloma diagnosed three years prior, for which he was receiving ongoing chemotherapy. He had no history of hypertension or diabetes mellitus, and there was no family history of cardiac disease.

On physical examination, the patient appeared clinically stable but showed signs of volume overload. Vital signs revealed a blood pressure of 120/80 mmHg, heart rate of 88 beats per minute, and oxygen saturation of 96% on room air. Cardiovascular examination revealed an elevated jugular venous pressure, bilateral fine crepitations on chest auscultation, and bilateral pitting edema extending up to the knees. The cardiac examination was notable for a regular rhythm with no audible murmurs, gallops, or friction rubs.

**Figure 1.**
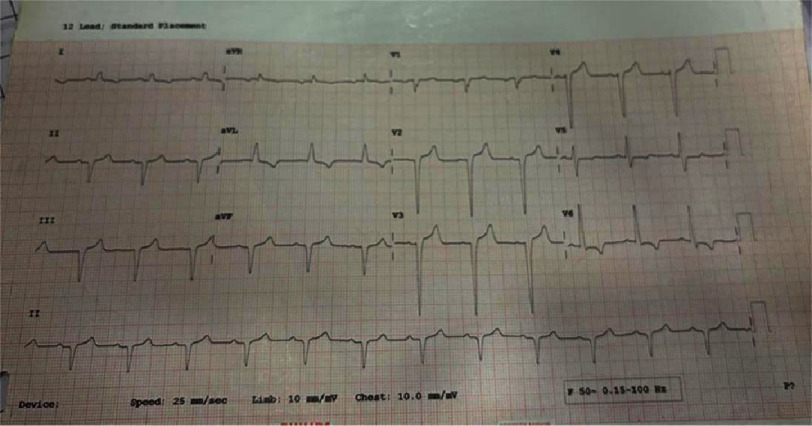
Standard -lead electrocardiogram demonstrating normal sinus rhythm with left axis deviation and left bundle branch block. Pathological Q waves are noted in the inferior leads (II, III, and aVF). Features suggestive of left ventricular hypertrophy with associated repolarization abnormality (strain pattern) are also evident.

Initial investigations included a 12-lead electrocardiogram (Figure 1) which demonstrated normal sinus rhythm with left axis deviation and left bundle branch block. Pathological Q waves were present in leads II, III, and aVF, along with left ventricular hypertrophy with strain pattern. These findings were particularly significant given the patient’s normotensive status and lack of traditional cardiovascular risk factors.

**Figure 2.**
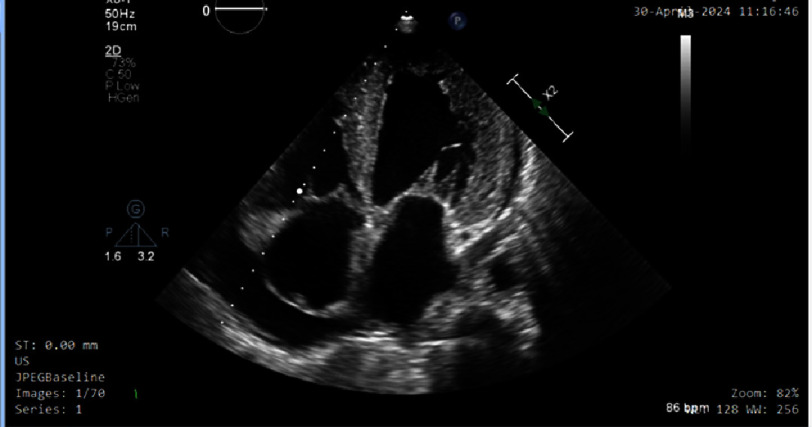
Two-dimensional transthoracic echocardiographic demonstrating concentric left ventricular hypertrophy.

**Figure 3.**
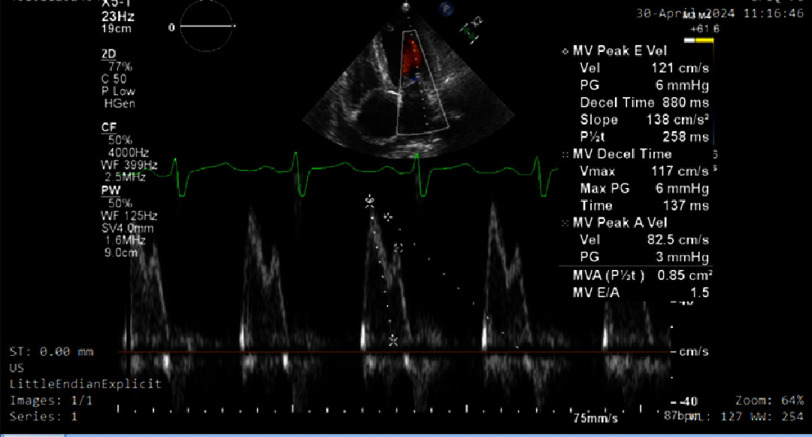
Pulsed wave Doppler recording across the mitral valve demonstrating peak early (E) diastolic velocity of 121 cm/s and late (A) diastolic velocity of 82.5 cm/s, yielding an E/A ratio of 1.5. This pseudonormalized filling pattern reflects elevated left ventricular filling pressures despite relatively preserved E and A velocities, a common finding in cardiac amyloidosis.

**Figure 4.**
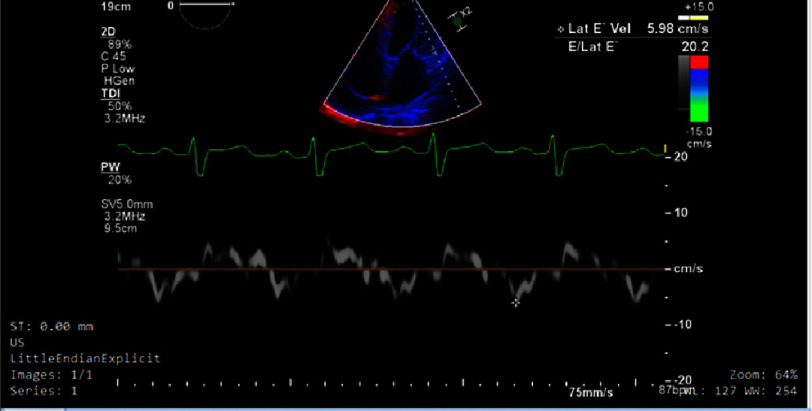
Tissue Doppler imaging (TDI) of the lateral mitral annulus demonstrating an early diastolic velocity (E’) of 5.98 cm/s, indicative of impaired myocardial relaxation.

**Figure 5.**
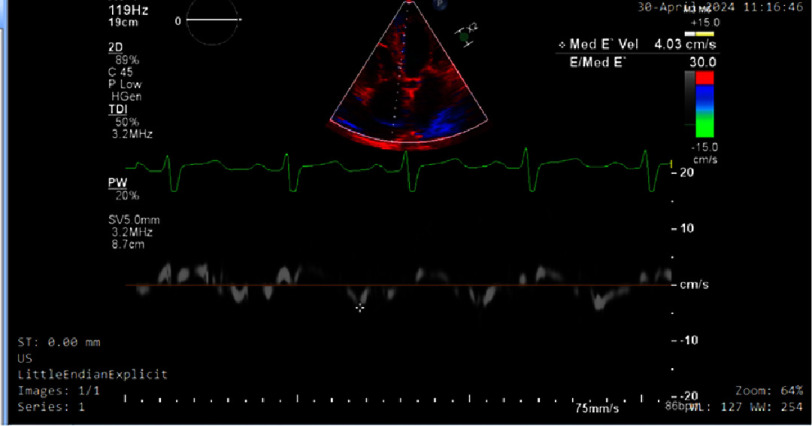
Tissue Doppler imaging (TDI) of the septal mitral annulus demonstrating an early diastolic velocity (E’) of 4.03 cm/s, yielding an elevated E/E’ ratio (>14 when combined with mitral inflow E 121 cm/s). This indicates markedly impaired myocardial relaxation and high left ventricular filling pressures, strongly supportive of grade III diastolic dysfunction in cardiac amyloidosis.

Transthoracic echocardiography (Figure 2) revealed moderate concentric left ventricular hypertrophy with moderately depressed left ventricular systolic function (ejection fraction 40%). Severe grade III diastolic dysfunction (Figures 3, 4 and 5) was evident, characterized by restrictive filling pattern. The combination of left ventricular hypertrophy in a normotensive patient without left ventricular outflow tract obstruction raised strong suspicion for infiltrative cardiomyopathy.

**Figure 6.**
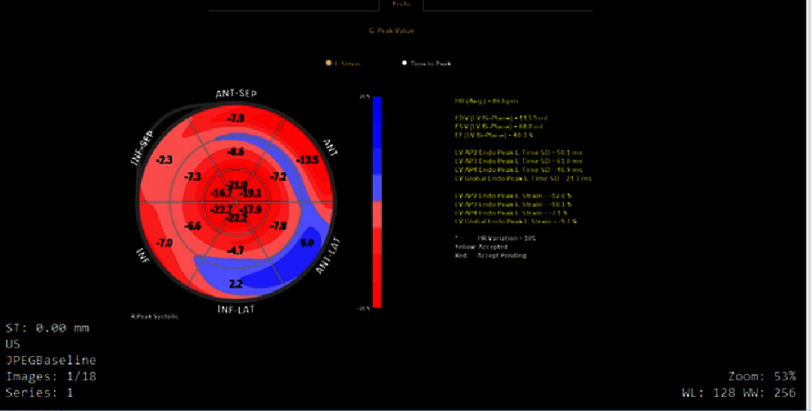
Speckle-tracking echocardiographic global longitudinal strain (GLS) bull’s-eye plot demonstrating severely reduced myocardial strain with a calculated GLS of −9.7%. The segmental strain map shows marked reduction in basal and mid-ventricular longitudinal deformation with relative preservation of apical strain, producing the characteristic apical sparing (“cherry-on-top”) pattern that is highly suggestive of cardiac amyloidosis in the appropriate clinical setting.

Given the clinical presentation and echocardiographic findings suggestive of cardiac amyloidosis, global longitudinal strain analysis (Figure 6) was performed. The GLS was markedly reduced at −9.7%, with characteristic regional strain distribution showing severely reduced strain values at the basal and mid-ventricular segments while apical strain remained relatively preserved. This pattern created the pathognomonic “cherry-on-top” appearance, which significantly enhanced the diagnostic confidence for cardiac amyloidosis.

Laboratory investigations supported the diagnosis, revealing elevated serum free light chains (kappa: 146 mg/L, lambda: 97.8 mg/L) with an abnormal kappa/lambda ratio of 1.49, high-sensitivity troponin T of 44 ng/L, NT-proBNP of 3106 pg/mL (corresponding to initial GLS −9.7%), and markedly elevated beta-2 microglobulin of 13,348 ng/mL indicating active disease. Serum creatinine was elevated at 2.3 mg/dL, consistent with impaired renal function commonly associated with multiple myeloma due to light chain cast nephropathy. Serum protein electrophoresis demonstrated a monoclonal spike with M-band prominence, and immunofixation electrophoresis confirmed the presence of monoclonal gammopathy. Renal biopsy performed to assess for systemic amyloidosis revealed AL amyloidosis with Congo red positive deposits and characteristic apple-green birefringence under polarized light microscopy. Electron microscopy confirmed the presence of randomly oriented non-branching fibrillary structures measuring 9–12 nm in diameter, consistent with amyloid deposits.

The patient was managed with guideline-directed medical therapy for HFrEF, including ACE inhibitor (tablet ramipril 2.5 mg daily), beta-blocker (tablet metoprolol succinate 50 mg once daily), SGLT2 inhibitor (tablet dapagliflozin 10 mg daily), and loop diuretics, alongside continuation of multiple myeloma treatment with BD regimen (bortezomib-dexamethasone). The BD regimen proved safe and appropriate in cardiac AL amyloidosis, as bortezomib rapidly reduces pathogenic light chain production driving myocardial infiltration without inherent cardiotoxicity; weekly subcutaneous bortezomib (1.3 mg/m^2^ on days 1, 8, 15, 22 of 35-day cycles) with reduced-dose dexamethasone (20 mg on dosing days) minimized risks of fluid retention, hypertension, and arrhythmias in the setting of HFrEF.

**Figure 7.**
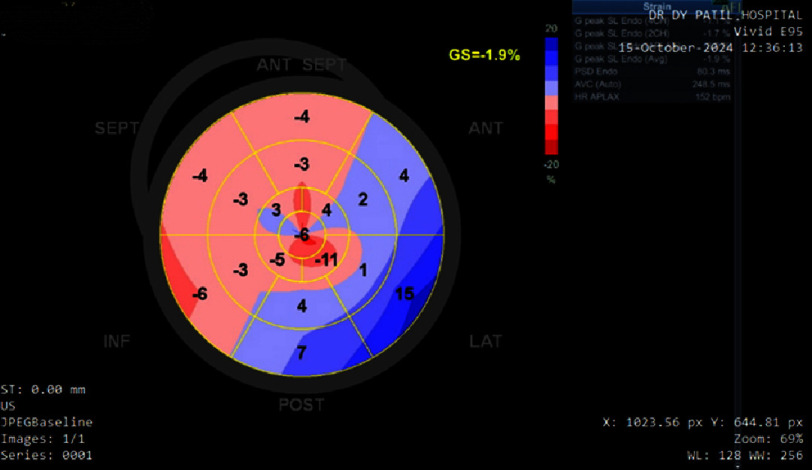
Follow-up speckle-tracking transthoracic echocardiographic global longitudinal strain (GLS) bull’s-eye plot demonstrating severely impaired myocardial strain with a calculated GLS of −1.9%. The segmental strain map shows diffuse reduction in longitudinal deformation with loss of the previously observed apical sparing pattern, consistent with interval progression of myocardial involvement compared with the baseline study.

At six-month follow-up, despite optimal guideline-directed medical therapy—including uptitration of ramipril to 5 mg daily—repeat echocardiography with strain analysis revealed further deterioration in cardiac function. Despite effective hematologic treatment of the underlying plasma cell dyscrasia with bortezomib-dexamethasone (BD) regimen, global longitudinal strain (GLS) worsened significantly to −1.9% (Figure 7), with NT-proBNP rising to 19,701 pg/mL, indicating progressive cardiac amyloidosis. This dramatic decline in GLS values correlated with the patient’s clinical deterioration and poor functional status, demonstrating the prognostic value of serial strain measurements in monitoring disease progression.

## Discussion

Our case demonstrates the complex interplay between multiple myeloma and cardiac amyloidosis, highlighting global longitudinal strain (GLS)’s crucial role in diagnosis and monitoring. Initial GLS of −9.7% (Figure 6) with apical sparing provided strong diagnostic evidence, consistent with the “cherry-on-top” pattern’s 93% sensitivity and 82% specificity per Phelan et al^6^. This landmark study established strain-based diagnosis as standard.

The apical sparing pattern aligns with large-scale validation studies. Pagourelias et al. showed the ejection fraction to GLS ratio (EFSR) best discriminated cardiac amyloidosis from hypertrophic cardiomyopathy and hypertensive heart disease in 100 patients^10^. Our patient’s EF 40% with GLS −9.7% created pathognomonic dissociation typical of infiltrative cardiomyopathy.

The diagnostic accuracy of GLS has been further enhanced by the development of multiparametric scoring systems. Boldrini et al. developed comprehensive echocardiographic scores for cardiac amyloidosis diagnosis, showing that a multivariable logistic regression model incorporating relative wall thickness, E/e’ ratio, longitudinal strain, and tricuspid annular plane systolic excursion had excellent diagnostic performance in AL amyloidosis (AUC: 0.90; 95% CI [0.87–0.92])^11^. Our patient’s clinical presentation with elevated E/e’ ratio, reduced longitudinal strain, and structural abnormalities would likely achieve a high diagnostic score using these validated algorithms.

Integration of strain parameters with cardiac biomarkers improves diagnostic accuracy in AL amyloidosis. Nicol et al. proposed a diagnostic score combining global longitudinal strain, apical-to-basal strain ratio, and troponin T, demonstrating high sensitivity and specificity for cardiac involvement^12^. Our patient’s markedly reduced GLS and elevated cardiac biomarkers would meet multiple criteria in this scoring system.

The association between multiple myeloma and AL amyloidosis in our case reflects well-established epidemiological relationships. Amyloid light-chain amyloidosis occurs in approximately 10% of patients with multiple myeloma, with cardiac involvement seen in 50–70% of those cases^3^. Our patient’s three-year history of multiple myeloma preceding cardiac presentation is consistent with temporal patterns described in large registries. Ríos-Tamayo et al. studied 158 patients with AL amyloidosis and found that patients with concurrent multiple myeloma had poorer median overall survival than AL-only patients (35.5 vs. 52.6 months), though this difference was not statistically significant^13^. The prognosis in AL amyloidosis is dominated by cardiac involvement, making early detection and monitoring crucial.

The dramatic deterioration in GLS from −9.7% to −1.9% over six months represents one of the most severe progressions documented in the literature. This finding is consistent with studies showing that GLS provides incremental prognostic value beyond established clinical and biochemical markers. Lee Chuy K et al. studied patients with advanced AL amyloidosis (Mayo stage III or IV) and found that GLS was the strongest predictor of survival compared to other validated risk factors including troponin, BNP, and left ventricular ejection fraction^14^. The authors concluded that GLS should be considered a standard parameter alongside serum cardiac biomarkers when evaluating prognosis in advanced disease.

The utility of GLS extends beyond diagnosis to monitoring treatment response and disease progression. Minamisawa et al. analyzed patients in the APOLLO trial and demonstrated that patisiran treatment attenuated deterioration of left ventricular global longitudinal strain in patients with hereditary transthyretin amyloidosis^15^. While our patient had AL amyloidosis rather than ATTR, the principle of using serial GLS measurements for monitoring remains applicable. The worsening GLS in our case, despite optimal medical management, suggests progressive amyloid deposition and poor treatment response.

The restrictive cardiomyopathy pattern observed in our patient, characterized by severe diastolic dysfunction with preserved wall motion in early stages, is typical of cardiac amyloidosis. The combination of clinical presentation, characteristic ECG changes showing left ventricular hypertrophy in a normotensive patient, and the pathognomonic strain pattern provided a compelling diagnostic picture even before tissue confirmation.

Our case also highlights the importance of early recognition and diagnosis in the era of emerging therapies. Despite optimal management of both underlying multiple myeloma and heart failure, the dramatic progression evidenced by GLS deterioration suggests that earlier intervention might have been beneficial. Contemporary studies emphasize that AL amyloidosis represents a poor prognostic factor for multiple myeloma, reinforcing the need for vigilant cardiac surveillance in patients with plasma cell disorders^16^.

GLS integration into routine cardiac assessments for multiple myeloma patients is warranted as per our experience and literature. Longitudinal strain has evolved from simple echocardiographic parameter to multipurpose biomarker for diagnosis, risk stratification, and decision-making in AL cardiac amyloidosis—a key advance^17^. Our case supports routine GLS use in this high-risk group, for both diagnostic and prognostic purposes, particularly in the context of emerging therapeutic options that may be most effective when administered before advanced cardiac dysfunction develops.

## Limitations

A limitation of our case report is the absence of endomyocardial biopsy and cardiac MRI, the latter not performed due to the patient’s deranged renal function tests (serum creatinine 2.3 mg/dL). Despite this, comprehensive echocardiographic evaluation revealing the characteristic “cherry-on-top” GLS pattern (−9.7% initially, deteriorating to −1.9%) combined with supportive clinical findings (refractory heart failure, LVH in a normotensive patient) and renal biopsy confirmation of AL amyloidosis provided sufficient diagnostic certainty for cardiac involvement. This non invasive multimodal approach aligns with studies demonstrating that such assessments often negate the need for invasive endomyocardial biopsy in systemic AL amyloidosis with strong imaging evidence, as shown by Aljama et al. where NT-proBNP plus echocardiography diagnosed cardiac involvement in most patients without requiring biopsy^18^.

### What we have learned?

Clinicians should maintain a high index of suspicion for cardiac amyloidosis in patients with multiple myeloma presenting with heart failure and unexplained left ventricular hypertrophy. Global longitudinal strain should be routinely incorporated into echocardiographic assessment in this population, as the characteristic apical sparing pattern provides an important noninvasive diagnostic clue. Serial GLS evaluation is a practical tool for monitoring disease progression and prognostication. Early identification of cardiac involvement and close collaboration between cardiology and hematology teams are essential to guide management and improve outcomes.
